# Wheat Crop under Waterlogging: Potential Soil and Plant Effects

**DOI:** 10.3390/plants12010149

**Published:** 2022-12-28

**Authors:** Isabel P. Pais, Rita Moreira, José N. Semedo, José C. Ramalho, Fernando C. Lidon, José Coutinho, Benvindo Maçãs, Paula Scotti-Campos

**Affiliations:** 1 Instituto Nacional de Investigação Agrária e Veterinária, I.P., Quinta do Marquês, Av. República, 2784-505 Oeiras, Portugal; 2 GeoBioTec Research Center, Faculdade de Ciências e Tecnologia, Campus da Caparica, Universidade Nova de Lisboa, 2829-516 Caparica, Portugal; 3 PlantStress & Biodiversity Lab, Centro de Estudos Florestais (CEF), Instituto Superior Agronomia (ISA), Universidade de Lisboa (ULisboa), Quinta do Marquês, Av. República, 2784-505 Oeiras, Portugal; 4 Earth Sciences Department, Faculdade de Ciências e Tecnologia, Campus da Caparica, Universidade Nova de Lisboa, 2829-516 Caparica, Portugal; 5 Instituto Nacional de Investigação Agrária e Veterinária, I.P., Estrada Gil Vaz, Ap. 6, 7350-901 Elvas, Portugal

**Keywords:** flooding, roots, gas exchanges, oxidative stress, *Triticum*

## Abstract

Inundation, excessive precipitation, or inadequate field drainage can cause waterlogging of cultivated land. It is anticipated that climate change will increase the frequency, intensity, and unpredictability of flooding events. This stress affects 10–15 million hectares of wheat every year, resulting in 20–50% yield losses. Since this crop greatly sustains a population’s food demands, providing ca. 20% of the world’s energy and protein diets requirements, it is crucial to understand changes in soil and plant physiology under excess water conditions. Variations in redox potential, pH, nutrient availability, and electrical conductivity of waterlogged soil will be addressed, as well as their impacts in major plant responses, such as root system and plant development. Waterlogging effects at the leaf level will also be addressed, with a particular focus on gas exchanges, photosynthetic pigments, soluble sugars, membrane integrity, lipids, and oxidative stress.

## 1. Introduction

Waterlogging is one of the main abiotic stresses affecting crop productivity worldwide [1]. However, due to climate change, the number of flooding events has increased in recent decades, as a result of more severe and unpredictable rainfall [2,3]. Overall waterlogging affects 10 to 15 million ha of wheat cultivated area, causing annual yield losses of 20 to 50% [4]. Global warming will likely boost extreme climatic phenomena, extending the risk of flooding also to new areas [5].

Water plays a vital role in plant growth, being crucial in plants’ interaction with the environment [6,7]. However, excess water in the soil may cause changes in soil properties such as reduced soil oxygen availability, disrupting its diffusion to plant tissues [8] and leading to hypoxic/anoxic conditions [9]. The effects of waterlogging depend on a number of variables, including the depth and duration of waterlogging [10], the crop’s developmental stage [11,12], and the weather conditions [10].

In a waterlogged environment, oxygen deficiency can limit root growth and ultimately lead to root death. In such conditions, energy-dependent processes such as water and nutrient absorption and transport to the shoot are impaired, compromising plant growth and development [10,13,14,15] as well as final yield [15,16,17]. In addition, decreases in leaf nitrogen content, leaf water potential, stomatal conductance, CO_2_ assimilation rate and photosynthesis, as well as accelerated leaf chlorosis and senescence may also be observed [15,17]. The down-regulation of the photosynthetic machinery in waterlogging sensitive plants results in the excessive production of reactive oxygen species (ROS) causing severe oxidative damage and degradation of cellular structures, interfering with normal metabolism [10,18]. ROS cause lipoperoxidation phenomena resulting in membrane injury, protein degradation, enzyme inactivation, damage to nucleic acids and, eventually, cell death [19,20,21,22].

Wheat is the third most cultivated cereal crop, supplying approximately 20% of the world’s energy and protein requirements [23]. In 2020, wheat global production reached a productivity of ca. 3.5 t ha^−1^ [24]. Taking into account predictable population growth and climate change scenarios, it is necessary to increase wheat productivity [23], and ensure production stability, two essential components of food security.

We will address some issues related to waterlogging effects in wheat crops, namely soil redox potential, pH, nutrient availability, and electrical conductivity, as well as their impacts in the root system and plant development. We will also focus on major leaf responses related to photosynthetic activity and membrane integrity under oxidative stress conditions.

## 2. Main Waterlogging Effects in Soil

Waterlogging can occur whenever soil moisture levels go beyond the field capacity. In such scenarios, excess water saturates the soil pores in the presence of a very slim water layer on the soil surface, or even in its absence [12]. This abiotic stress has a negative effect on the majority of terrestrial plants, limiting crop yield. Changes in the physical, chemical, electrochemical, and biological properties of the soil (Table 1) can lead to a decrease in root biomass, hampering vegetative development [10,25,26] and inducing plant organs senescence [27,28].

In a waterlogged soil, all pores are filled with water, changing the ideal solid:pore material (50:50) and soil:air volume (75:25) ratios, which have implications for the plant’s physiological performance [29]. The atmosphere contains 21% oxygen, while the soil should have a concentration of at least 10% [30]. In cultivated soil, the dissolved [O_2_] is around 0.23 mol m^−3^, whereas in waterlogged areas, the level is less than 0.05 mmol m^−3^ [31]. Under normal conditions, the soil atmosphere is rich in CO_2_ and deficient in O_2_ [29] due to the aerobic respiration of roots and microorganisms. However, good aeration enables rapid O_2_ intake and CO_2_ output, providing an adequate amount of oxygen to meet plant nutritional requirements and allow its development [29]. When waterlogging is established, gas exchanges between soil and atmosphere almost stop as gas diffusion in water is 10^4^ times slower than in air [32]. Plant roots respiration and microbial activity use the oxygen trapped in the soil, promoting a hypoxia/anoxic situation in the rhizosphere [9], resulting in both insufficient O_2_ levels and toxic CO_2_ concentrations in soil.

Significant changes in redox potential (Eh) can be caused by soil waterlogging. Eh is the most important physicochemical parameter for the evaluation of the oxidation or reduction level of a flooded soil. In general, soil [O_2_] is inversely proportional to the Eh. In flooded soils, oxygen deficiency leads to biological reduction processes and a decrease in Eh. According to Søndergaard [33], Eh will be approximately 250 mV and 0 mV for [O_2_] values of ca. 10% and 1–2%, respectively. Under optimal aeration conditions, cultivated soils have Eh values between +300 and +500 mV [34], with +400 to +450 mV apparently being ideal [35]. In plants roots, the change from aerobic to anaerobic metabolism occurs when [O_2_] < 1% [33]. In waterlogged soils, Eh can reach values between −300 and +100 mV [35], explaining why growth decreases dramatically in plants susceptible to this stress.

The optimal pH range for the vast majority of cultivated plants is 6.5 to 7 and it is considered ideal for nutrient availability. Nonetheless, values between 5.5 and 8 still provide favorable growth conditions [35]. In waterlogged soils, pH tends toward neutrality, with increases in acidic soils and decreases in alkaline ones [35,36]. Soil pH strongly influences the solubility, mobility, and bioavailability of nutrients and potentially toxic elements, which in turn affects their uptake by plants [37]. Soil acidity is associated with Mo, P, Mg, and Ca deficiency. At a low pH, other elements such as Al, Mn, and Fe may become more available while Al, Fe, and Mn may reach toxic levels to plants [38]. Alkaline soils, on the other hand, are typically deficient in Co and Zn and show less P availability since Ca can bind to it. In these conditions plants tend to be underdeveloped, depicting poor growth and lower yield [10,39,40,41,42].

Nitrogen (N), an essential element for plant growth and one of the most crucial yield-limiting nutrients, is typically extracted from the soil in the inorganic forms of ammonium (NH_4+_) or nitrate (NO_3_^−^). In addition to the amount of nitrogen available in the soil, the form in which it is present can influence plant physiological and metabolic processes, such as nutrient uptake, enzyme activity, photosynthesis and respiration rate, water balance, and signaling pathways [43,44]. Waterlogging significantly reduces gas diffusion, leading to an increase in NH_4+_ in the soil. Although this ion is an intermediary in numerous metabolic reactions, when it is the only source of N, it may induce a strong inhibition of K uptake, an essential nutrient that is also involved in several important plant physiological processes [44]. Wheat grows preferentially on NO_3−_ nutrition. In waterlogged soils, substantial decreases (15–20%) in wheat growth and yield were reported [10], possibly due high NH_4+_ levels.

Electrical conductivity (EC) is a good indicative of soil quality [45], as it correlates with the concentration of NO_3_^−^, sulphate (SO_4_^2−^), NH_4+_, K, S, and Cl, as well as the soil’s nutrient availability. Significant changes in EC can be caused by soil waterlogging. At the onset of that stress, soil EC tends to increase, reach its maximum, and then decrease to stable values. This rise is due to the mobilization of Fe^2+^ and Mn^2+^, the accumulation of NH_4+_, HCO_3_, and RCOO-, as well as the displacement of cations adsorbed on colloids by Fe^2+^, Mn^2+^, and NH_4+_ [46]. Due to nutrient leaching, the soil may be less fertile after a flood [47]. Low EC values may indicate nutrient deficiency and, if less than 0.10 dS m^−1^, soil deterioration [22].

## 3. Plant Responses to Waterlogging

Plants response to waterlogging is highly dependent on a number of factors, such as the depth and duration of exposure and the plant developmental stage [11,41,48,49], among others.

Several authors have identified the wheat reproductive phases, including the stem elongation stage to anthesis and post-anthesis, as the plants’ most sensitive time to waterlogging stress [11,14,50]. Wu et al. [49] found the greatest negative impact at the seedling stage while Pampana et al. [12] observed no differences at the three and four leaf stages. Ding et al. [51] reported wheat yield reductions by 9 to 15% when waterlogging was imposed at the seedling, jointing, and tillering stages and decreased leaf area by 10% and 29% at anthesis and at the milk-ripe stage, respectively. At the tillering stage, reduced spike and grain numbers were reported, whereas waterlogging at booting decreases grain weight [49]. The highest tolerance to waterlogging was found in the period after anthesis followed by the jointing stage [14].

Under natural conditions, waterlogging depths can change, influencing the severity of plant damage. Depending on how deeply water penetrates the soil, total or partial waterlogging can be considered. Total waterlogging occurs when water is present from the soil’s bottom to its surface, affecting all plant organs below ground. On the other hand, partial waterlogging occurs when water does not reach the soil surface and only affects a portion of the root systems [9]. According to Malik et al. [28] bread wheat tillering was reduced by 24, 45, and 62% when the water level was 20, 10, and 0 cm below the soil surface, with decreases of 39, 58, and 73% (respectively) in length of adventitious root main axes per plant.

The duration of waterlogging events has a significant effect on the damage caused to crops. Overall, the longer the duration, the greater the negative effects in plants [52,53]. Reported impacts of waterlogging include changes in plant photosynthesis, respiration, transpiration, and antioxidative system, enhanced organs senescence and reduced accumulation and remobilization of photosynthetic products, that ultimately result in decreased yield components such as spikes number, kernels per spike, and kernel weight [4,10,13,14,52,54]. Lower soil O_2_ availability is the primary cause of observed negative effects [10,55,56], but anatomical, morphological, or physiological changes may help to mitigate the effects of such deficiency in plants [57].

### 3.1. Roots

**Figure 1:**
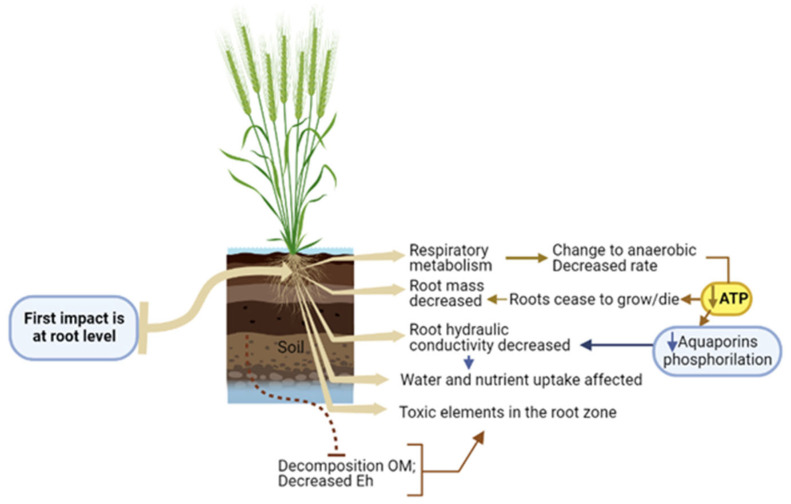
Major effects of waterlogging occurring at root level. Figure elements created using biorender.com (accessed on 15 December 2022).

Roots are essential organs for water and nutrients uptake, photoassimilates storage, anchorage, mechanical support, and rhizosphere interface [58,59]. To accomplish this, roots require energy from cellular respiration [60]. Waterlogging conditions primarily affect the plant at the root level, where the initial responses occur [61] (Figure 1).

Root damage causes severe shoot growth impairments [10,15,62]. Arrested root growth and root death significantly decrease seminal root dry mass [10,15]. Under waterlogging conditions low [O_2_] in the rhizosphere enhances anaerobic respiration [63], resulting in lower ATP production [64]. Energy deficit at the root level impairs aquaporins phosphorylation, which regulates cell water flux, compromising water and nutrient absorption due to a drastic reduction in root hydraulic conductivity [15,65]. Additionally, the drop of soil Eh may increase the availability of Mn_2+_ and Fe_2+_ to toxic levels and their accumulation in the roots. Organic acids and other potentially toxic metabolites produced by decomposition of organic matter in anoxic soils may increase in saturated soils. Anaerobic respiration can result in accumulation of lactic acid, ethanol, aldehydes, among others, and in ROS production, namely hydrogen peroxide, which can also cause cellular damage [31,66]. In rice, organic acid toxicity inhibits root respiration, reduces nutrient uptake, increases suberization and/or lignification of outer cell walls, and can cause root occlusions [67]. In barley, adverse effects of organic acids on K^+^ fluxes in roots were reported [68]. Endogenously-produced CO_2_ and ethylene can also adversely affect roots, as pH in root cells can became more acidic due to excess CO_2_, and high ethylene can inhibit root extension [10,32,69].

Some plants tolerant to hypoxia (low [O_2_]) or anoxia (no O_2_) can develop morphological adaptations to compensate for the lack of O_2_ in the root zone [28,70]. Adventitious root formation from the stem or branches is a common response [70] that promotes gas transport, as well as nutrient and water uptake, during waterlogging, significantly enhancing plant survival and productivity [71]. These roots are able to take up and transport O_2_, making it available to submerged roots [72]. According to Wiengweera and Greenway [73], in wheat plants subjected to waterlogging, adventitious roots absorbed more efficiently P and K than seminal roots. Root architecture can also differ in wheat genotypes, leading to different root distributions in the soil [74]. In flooded areas, shallower root systems may be beneficial in oxygen uptake, since upper soil layers usually have a higher O_2_ concentration than deeper ones [75,76].

Under waterlogging stress, ethylene accumulation can also activate programmed cell death of root cortical cells, inducing the formation of aerenchyma in adventitious roots. Aerenchyma development in wheat can boost plant tolerance and survival under conditions of waterlogging and oxygen deprivation [77], since it increases energy availability and reduces hypoxic stress [72,78]. This specialized parenchymal tissue has interconnected, large, gas-filled intercellular spaces that provide a low-resistance pathway, hence enhancing gas diffusion between the roots and shoot [72,77]. Furthermore, aerenchyma can discharge CO_2_ and toxic volatile substances from submerged tissues [31]. However, the internal O_2_ movement to the apex, which enables root expansion, has a limited extent, and adventitious root growth does not fully compensate seminal root loss. In response to waterlogging, the root:shoot ratio in wheat can decrease from 0.4 to 0.2, reflecting the stronger decrease of root dry mass (up to 62%) when compared with shoot (up to 33%) [10].

### 3.2. Shoot Development and Productivity

**Figure 2:**
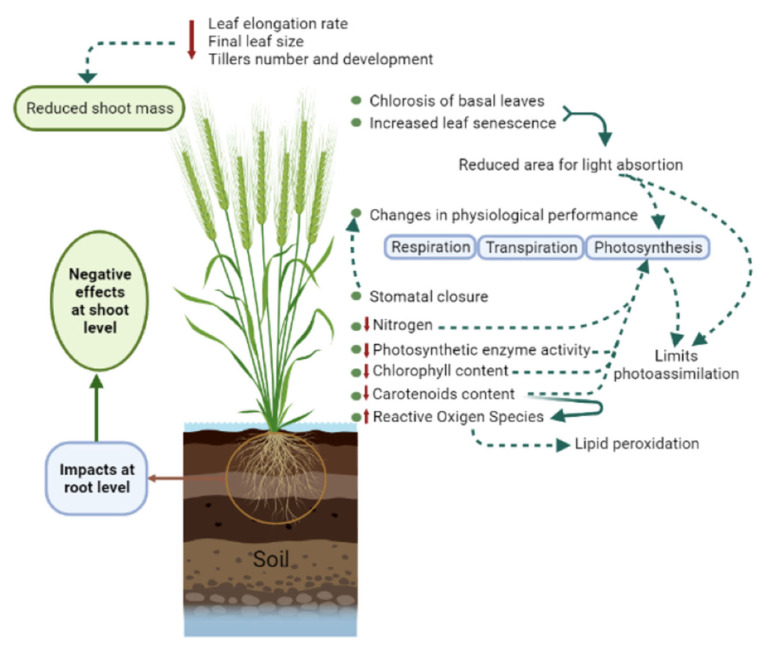
Effects of waterlogging at shoot level. Figure elements created using biorender.com (accessed on 15 December 2022).

Water uptake by the roots and transpiration from the leaves allow plants growth but, when subjected to waterlogging, susceptible genotypes may present severe impairments of *s*ome key metabolic activities, such as photosynthesis, respiration and transpiration. The detrimental effect on these processes causes severe energy deficiency, poor growth, and enhanced leaf/organs senescence, decreasing the accumulation and remobilization of photoassimilates and hence grain yield [15,64,79] (Figure 2).

Several authors report that waterlogging significantly inhibits the growth of aerial parts of the plants, mostly due to a reduced leaf elongation rate, resulting in smaller leaves, but also due to a decrease in the number of tillers and tillers impaired development [10,16,28,62,80,81]. According to Malik et al. [15], the imposition of 3–21 days of waterlogging on 3-week-old plants, followed by 21 or 7 days of recovery, decreased the shoot mass by 43% to 72%, in comparison to well-drained plants over the same period. Herzog et al. [10] reported a 67% reduction in average shoot dry mass of wheat plants grown in waterlogged soil. This decline, together with the overall wilting of the plant and the senescence of the basal leaves, significantly reduces the area available for light absorption and limits photoassimilation. In bread wheat, chlorotic dry mass increased from 8–21% in non-waterlogged plants to 33–70% in 14-days-waterlogged plants [82]. During the recovery period this trend was maintained, with control plants exhibiting lower chlorotic dry mass values (14–36% and 18–43% at 7- and 14-days recovery, respectively) than treated plants (37–95% and 49–100% for the same recovery periods).

### 3.3. Impacts in Physiological Performance

#### 3.3.1. Gas Exchanges and Sugars Metabolism

Changes in respiration and photosynthesis (Pn) are caused by the high sensitivity of photosynthetic processes to stressful conditions [20,83], frequently used as indicators of productivity under stress conditions. In plants susceptible to waterlogging, physiological activities are drastically reduced and may cause cell death, whereas tolerant plants depict less severe effects or even an improvement in the response of some parameters [28].

As a result of impaired root function, a reduction in shoot physiological performance may occur in wheat plants subjected to waterlogging [10,17,84]. Stomatal closure, reduced transpiration, and photosynthesis inhibition are typical responses to this environmental stress [10,17,84]. Stomatal conductance (gs) is one of the primary factors influencing photosynthesis [85], with a significant effect on photosynthetic rates under waterlogged conditions [10,28]. Stomatal closure and (gs) decrease allow a down-regulation of leaf transpiration [10]. However, this also lowers internal CO_2_ concentration (Ci), which in turn limits carbon fixation, reducing photosynthesis and increasing respiration, negatively affecting plant production [28,86]. However, photosynthetic rates can also decrease due to non-stomatal factors such as chlorophyll degradation and decreased chlorophyll synthesis, resulting in leaf senescence and yellowing [10,57,80]. Damage to the photosystem II by ROS, decreased photosynthetic enzyme activities, and low nitrogen content exacerbated the decline in photosynthetic activity and the detrimental effects of waterlogging [10,57,80,87]. Through Pn, plants convert carbon dioxide and water into sugars, which are their principal source of energy for different cellular activities [88]. Waterlogging can lead to changes in Pn and respiration, with consequences for sugar metabolism and energetic balance [89]. Lower Pn rates reported at the onset of waterlogging may be related to the accumulation of sugars in the leaves rather than to stomatal closure [10,15]. The accumulation of sugars in leaves of waterlogged wheat plants has been reported [10,15,63].

At the onset of waterlogging, sugars accumulation may result from root hypoxia, which inhibits the rapid growth of both the root and the aboveground portion of the plant. Under such conditions, sugar production in the leaves exceeds its consumption [10,15,28]. Concurrently, constraints in the root system diminish the phloem transport capacity of the roots [10,15], which also contributes to the accumulation of photoassimilates in the leaves. The resulting sugar overproduction, together with a reduced ability for phloem transport in hypoxic roots, leads to further decrease in Pn as a negative feedback of carbohydrate accumulation [10].

In long-term flooding, plants experience energy and carbohydrate deprivation due to reduced photosynthesis and aerobic respiration. Therefore, the initial carbohydrate reserves may be a crucial factor in tolerance to hypoxia/anoxia, as the plant must utilize its stored glucose reserves to maintain metabolic activity under anaerobic conditions [89,90]. Additionally, sugars are also involved in plant stress responses and adaptation, contributing to the stabilization of membrane structures and maintenance of cell turgor through osmotic adjustment and osmoprotection [91].

#### 3.3.2. Chlorophylls and Carotenoids

Photosynthetic pigments are fundamental molecules in the photosynthetic process, their primary function being light absorption and the production of reducing compounds [31,92]. Changes in pigment content and composition have a direct effect on the photosynthetic rate. Chlorophylls are essential for the conversion of light radiation into chemical energy. They are strictly associated with photosynthetic efficiency and, therefore, with plants growth and environmental adaptability [31,92]. Chlorophyll a is present at the reaction centers of both photosystems (PSI and PSII), whereas chlorophyll b is the most important accessory light-absorbing pigment in light-harvesting complexes.

Several authors observed decreases in chlorophyll content with waterlogging. At the emergence stage, a 10-day’s stress induced chlorophyll reductions between 15 and 33% in four wheat genotypes [93]. At the tillering stage, reductions from 41% to 61% were observed in six wheat varieties subjected to 28 days waterlogging [80].

Carotenoids have several functions in plant metabolism. As photosynthetic pigments, they are accessory antenna molecules, harvesting and transferring light energy to chlorophylls during the photosynthetic process. Carotenoids also play an important role in oxidative stress tolerance, contributing to protect the photosynthetic apparatus by scavenging ROS and repressing lipid peroxidation [20]. Waterlogging can affect the concentration of carotenoid pigments and several studies reported their reduction in wheat-susceptible plants [57,81,94]. However, in tolerant genotypes, the amount remained high [95]. Overall, the decline in carotenoid content is more severe in plants subjected to longer waterlogging periods. At the tillering stage, carotenoid contents decreases by 11–15%, 16–38%, and 29–67% after 7, 14, and 21 days of waterlogging, respectively [94]. The same study reported that 14 days of waterlogging at elongation stage caused a more severe carotenoid lowering (32–49%), highlighting different effects according to crop development phase.

#### 3.3.3. Chlorophyll Fluorescence

In the light-harvesting antenna, light photons are captured by chlorophyll and partially (ca. 2%) re-emitted as fluorescence [96]. Chlorophyll fluorescence is a reliable and sensitive tool to assess light-harvesting efficiency in plants [97,98], that complements information obtained through gas exchanges. Under stress conditions, this parameter can decrease, allowing a quantitative comparison of the stress responses, and indirectly providing information on leaf photosynthetic performance.

Maximum quantum efficiency of PSII (Fv/Fm) evaluates the proportion of functional PSII reaction centers. Reductions in this ratio can indicate damage to the photosynthetic apparatus which that may result in Pn decreases [48]. In wheat, declines in Fv/Fm ratio have been reported due to the imposition of waterlogging, indicating impairment of PSII [10,49,80] and consequently, a decreased use-efficiency of captured photon energy [48].

#### 3.3.4. Membrane Integrity and the Role of Lipid Composition

The ability to maintain membrane integrity under stressful conditions ensures cellular compartmentalization and the functioning of metabolic processes, being determinant to protoplasmic tolerance [83]. Waterlogging, as well as other biotic and/or abiotic stresses, can lead to changes at the membrane level, with structural impacts that affect membrane permeability, assessed through increased electrolyte leakage from cells [83,99,100,101], that may reflect severe membrane damage and low survival ability [93,102]. Therefore, membrane stability is frequently used as an indicator of tolerance or susceptibility to environmental stresses [83,103].

Lipids are fundamental plant macromolecules playing key roles in membranes structure, energy storage, and metabolic signaling [104,105,106,107]. In response to abiotic stresses, qualitative and quantitative changes may occur in of the lipid matrix compositions, such remodeling plasticity being crucial for maintenance of membrane integrity [99] significantly contributing to its functionality [66,107]. Hypoxia/anoxia can induce changes in membrane lipids, and it was shown that tolerant plants could increase the degree of unsaturation of membrane lipids, and also enhances lipids biosynthesis under such conditions [102,106].

Lipid remodeling influences the fluidity, integrity, and permeability of plant cell membranes through changes in the composition of lipid classes, the lengths of their carbon skeletons, or the saturation of their fatty acids [106,107]. Hypoxia treatment significantly altered the lipid composition of wheat, with tolerant genotypes exhibiting more efficient lipid remodeling, allowing the bilayer structure of membranes to be preserved during hypoxia stress [107]. Hypoxia reduced the phospholipids phosphatidylcholine (PC) and phosphatidylethanolamine (PE), but the PC:PE ratio increased in the tolerant genotype, thereby limiting the synthesis of non-bilayer membrane phases and conserving fluidity. Non-susceptible plants exhibited considerable increases in phosphatidylglycerol (PG) and phosphatidic acid (PA) as a result of hypoxia. Despite differences in PE content across tolerant and susceptible genotypes, hypoxia-induced alterations followed a similar pattern, suggesting that PE had no contribution to hypoxia tolerance. Several authors have found changes in the glycolipids monogalactosyldiacylglycerol (MGDG) and digalactosyldiacylglycerol (DGDG) in response to flooding [106]. After 4 days of exposure to hypoxia, Xu (2019) [107] observed a 31.6% and 20% decrease in MGDG concentrations in sensitive and tolerant genotypes, respectively. DGDG content was unchanged by hypoxia in the sensitive genotype, whereas in the tolerant genotype, an increase of 25.3% was seen after 2 days of treatment, followed by a drop of 31.1% on the fourth day of hypoxia. Changes in glycolipids can have a significant effect on plants’ tolerance to waterlogging since MGDG is essential for photosynthetic reactions and DGDG is essential for maintaining the maximum efficiency of photosynthetic electron flow by altering PSI and PSII activity [107].

#### 3.3.5. Oxidative Stress

Although ROS are a normal product of plant cell metabolism, biotic and abiotic stresses are often accompanied by oxidative stress, which is characterized by an increase in intracellular ROS. ROS can be divided into free radicals (superoxide radical (O_2_**^•−^**), hydroxyl radical (OH**^•^**), perhydroxy radical (HO_2_**^•^**)) and non-radicals (singlet oxygen (^1^O_2_), hydrogen peroxide (H_2_O_2_)) [31]. When accumulated in mesophyll cells, their strong oxidizing activity can lead to lipoperoxidation and degradation of membrane lipids, and cause oxidative damage to proteins and DNA, resulting in severe cell injuries [22,31,108]. Carbon–carbon double bonds in lipids are preferential targets for ROS, meaning that cell membranes are rich in polyunsaturated fatty acids (PUFA), particularly abundant in chloroplasts, and are extremely susceptible to lipoperoxidation [109,110]. In waterlogging-susceptible plants, the downregulation of the photosynthetic machinery leads to excessive ROS generation within the leaf. Rapid chlorosis of basal leaves precedes premature leaf senescence caused by the remobilization of nitrogen to younger leaves [10,111]. Decreased chlorophyll content in the remaining leaves is an indicator of oxidative stress [88]. Excess water can increase ROS content many-fold higher than normal growing conditions, causing severe oxidative damage to plant cells [18]. This rise suggests the presence of lipoperoxidation events [112] and is commonly associated with greater concentrations of malonyldialdehyde (MDA), one of several lipid oxidation products [109]. Genotypes showing lower MDA levels under stress may be more resistant to oxidative stress [110]. ROS accumulation also causes a significant disruption to plant ionic homeostasis, directly influencing the functioning of various cation [113] and anion channels [114]. Wheat plants can overcome oxidative stress through activation of antioxidative defense systems involving enzymatic and non-enzymatic mechanisms to neutralize excessive ROS and reduce the extent of oxidative damage [1,18,19,20,54].

By removing, neutralizing, or scavenging ROS and their intermediates, antioxidant enzymes such as superoxide dismutase (SOD), catalase (CAT), glutathione reductase (GR), and ascorbate peroxidase (APX) disrupt the cascades of uncontrolled oxidation, converting ROS into harmless compounds [90]. Non-enzymatic antioxidants, such as reduced glutathione (GSH), ascorbic acid (AsA), carotenoids, and tocopherols, play a crucial role in membrane stabilization and cellular components protection. Several reports of increased antioxidant enzyme activity as a result of waterlogging, have also been reported in tolerant wheat plants [31,94].

### 3.4. Waterlogging Effects in Yield Components

Wheat is considered susceptible to waterlogging, and decreases in grain yield per plant have been widely reported under such stress [12,52,57,62,81]. Waterlogging for 30 days throughout sowing, seedling, flowering, and grain-filling reduced grain yield by 50–70% due to poor seed set and fewer spikes per unit area [52,115]. At the tillering stage, 21 days of waterlogging resulted in lower yield with value reductions from 37% to 60% [94]. In waterlogging-susceptible genotypes, reductions were also observed in the number of spikes per plant [116], the number of grains per spike [4], and the thousand kernel weight [117].

The emission, development, and survival of tillers are crucial features since they are directly proportional to the number of spikes per unit area [118], which has a straight effect on wheat yield [28]. Studies with different wheat genotypes subjected to waterlogging during the tillering phase revealed that a reduction in the number of tillers emitted was not always accompanied by a reduction in the number of fertile ones, indicating a good strategy of the plant to maintain production to cope with energy deficit [28,62,81]. If the number of fertile tillers remains unaltered, the decline in productivity results from a small contribution of fertile tillers to the formation of the final yield [118]. Yield decreases under stress were associated with reduced production [15,28] due to low tillers survival, fewer and smaller fertile tillers, and smaller grains [119,120,121]. A significant decrease in seed number per spike (2.0 to 78.8%) with increasing time of waterlogging periods (5 to 50 days), with the lowest value at the 50th day and the highest seed number per spike in control plants [52]. Ding et al., (2020) [51] reported decreases in single-spike yield (9%) and in kernels per spikelet (5%) in waterlogged wheat, although no changes were observed concerning spikelet fertility and spikelets per spike. The same trend was found by Alizadeh-Vaskasi (2018) [94], who reported reductions in kernels-per-spike and single-spike yield in wheat plants subjected to waterlogging at tillering and elongation stages.

### 3.5. Genetic Responses to Waterlogging

Adverse environments, such as waterlogged soil, may induce several changes at morphological and physiological levels in plants. To enhance waterlogging tolerance, a vast number of stress response genes are activated and essential functional proteins are synthesized [122,123]. Wei et al., (2021) [124] observed a considerable down-regulation of photosynthesis-related genes (e.g., *PsbQ*, *PsbO*, and *petF*) and light-harvesting chlorophyll protein complex genes (e.g., *LHCB1*, *LHCB3*, *LHCB5*, *LHCA1*, and *LHCA4*) in two wheat genotypes in response to waterlogging. Borrego-Benjumea et al., (2020) [125] observed the participation of genes involved in multiple metabolic pathways at the root level of barley, including glucose and nitrogen metabolisms. The down-regulation of genes involved in ROS detoxification, nitrogen, and amino acid metabolism was also identified in the same study. In wheat, Tong et al., (2021) [126] reported an enhanced expression of RBOH (Respiratory Burst Oxidase Homolog), which regulates ROS accumulation. As the mechanism of ROS production/scavenging is essential for the regulation of aerenchyma development in roots, genes implicated in this mechanism have been considered candidates for waterlogging tolerance [126]. Same authors also found multiple Quantitative Trait Locus (QTLs) for waterlogging tolerance features related to root fresh biomass (QRfbio.ua-1B-WGH), shoot fresh biomass (QSfbio.ua-1B-WGH), chlorophyll content (QSpadpost.ua-1B-WF and QSpad.ua-1D.5), and germination rate index (GRI-7A). The knowledge of the genetic pathways linked with this stress can be of great value to the molecular breeding of waterlogging-tolerant wheat.

## 4. Conclusions

The increased frequency and intensity of extreme weather events, such as waterlogging episodes, resulting from global warming, is one of the challenges to maintaining/improve wheat yield. Plant growth and development processes under waterlogging depend on morphological, physiological, and biochemical adaptation, and on the gene regulation modulating such traits. Identifying key traits underlying tolerance responses and understanding their roles in adaptation to waterlogging will contribute to develop more adapted wheat plants and to boost wheat yield and grain quality under a changing climate.

## Figures and Tables

**Table 1:** Effects of waterlogging on the physical, electrochemical, chemical, and biological properties of the soil. Elements of the figure were created using biorender.com (accessed on 15 November 2022).

Impacts in Soil Properties Due to Waterlogging		
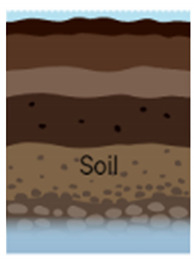	Physical	Changes in ideal solid:pore and soil:air volume ratios; Decreased [O_2_]; increased [CO_2_]; lowered diffusion coefficient for gases.
	Electrochemical	Decreased redox potential (Eh); Changes in soil pH and EC.
	Chemical	Changes in solubility, mobility, and bioavailability of nutrients and potentially toxic elements.
	Biological	Changes in microbial activity and in the nitrogen cycle (mineralization and immobilization of organic N).

## Data Availability

Not applicable.
